# Clinical expertise and cognitive reflection predict fewer misconceptions in Bayesian diagnostic reasoning

**DOI:** 10.1186/s12909-026-10764-z

**Published:** 2026-11-07

**Authors:** Markus H. Hefter, Nathan B. Hinkel, Andreas Hinkel

**Affiliations:** 1 Department of Psychology, Bielefeld University, Universitätsstr. 25, Bielefeld, 33615 Germany; 2 Department of Urology, Franziskus Hospital, Bielefeld, Germany

**Keywords:** Bayesian diagnostic reasoning, Medical knowledge, Clinical expertise, Cognitive reflection, Misconceptions

## Abstract

**Background:**

For many years, research has consistently confirmed that even medical experts fail to solve Bayesian diagnostic tasks. These tasks require correctly estimating a diagnostic test’s positive predictive value based on sensitivity, specificity, and the disease’s prevalence. Previous research has primarily focused on improving task presentation, for example by using natural frequencies or visual aids. The present study introduces the Medical Bayesian Performance Score (MBPS), a confidence-weighted measure that distinguishes confidently held misconceptions from missing concepts. Participants completed eight medical Bayesian diagnostic tasks and rated their confidence after each response. Using this approach, we examined whether medical knowledge, clinical expertise, and cognitive reflection are associated with performance in Bayesian diagnostic reasoning.

**Methods:**

In an online survey, we compared MBPS values via *t* tests along two independent variables: (a) medical knowledge (physician group: *N*_p_ = 30 vs. student control group: *N*_s_ = 56) and (b) clinical expertise within the physician group (specialists and senior physicians: *N*_e_ = 19 vs. resident physicians: *N*_r_ = 11). Furthermore, we analyzed correlations between MBPS values and Cognitive Reflection Test (CRT) scores.

**Results:**

Physicians did not achieve significantly higher MBPS values than the student control group. In contrast, specialists and senior physicians scored significantly higher than resident physicians. Across all groups, average MBPS values were negative, indicating that confidence-weighted incorrect responses outweighed confidence-weighted correct responses, a pattern consistent with misconceptions. Furthermore, higher CRT scores correlated significantly with higher MBPS values.

**Conclusions:**

The MBPS provides a complementary perspective to conventional accuracy measures by distinguishing misconceptions from missing concepts and linking Bayesian diagnostic reasoning research with conceptual change theory. Consistent with this perspective, our findings suggest that formal medical knowledge alone was not associated with fewer misconceptions, whereas greater clinical expertise and higher cognitive reflection were. Future research should evaluate evidence-based conceptual change interventions for medical education, such as specific refutation-based training, as a means of improving Bayesian diagnostic reasoning.

**Supplementary Information:**

The online version contains supplementary material available at https://doi.org/10.1186/s12909-026-10764-z.

## Introduction

What does it mean when a diagnostic test yields a positive result? Consider a prostate-specific antigen (PSA) screening test for the early detection of prostate cancer. Because the test manual states that approximately 86% of patients with prostate cancer receive a positive test result, it seems intuitively plausible to conclude that a positive result almost certainly indicates the presence of cancer. However, this conclusion is incorrect because the probability of disease given a positive test result is not equivalent to the probability of a positive test result given the presence of disease.

Correctly interpreting a diagnostic test requires integrating three fundamental parameters: disease prevalence, test sensitivity, and test specificity. These concepts are routinely introduced in standard textbooks on statistics for medical students, clinicians, and researchers [1–4]. *Prevalence* describes the baseline proportion of affected individuals within a population. *Sensitivity* denotes the probability of a positive test result given the presence of disease, whereas *specificity* denotes the probability of a negative test result given its absence. Together, these parameters determine a diagnostic test’s *positive predictive value* (PPV)—that is, the probability that an individual actually has the disease after receiving a positive test result.

A vivid real-world example illustrating the dependence of the positive predictive value on disease prevalence was provided by the German federal public health institute, the Robert Koch Institute (RKI), regarding the interpretation of SARS-CoV-2 antigen rapid tests [5]. Holding sensitivity (80%) and specificity (98%) constant, the RKI demonstrated how substantially the predictive value changes with disease prevalence. In a low-prevalence scenario (0.05%; 5 infected individuals per 10,000), the PPV was only 2.0%, indicating that 98% of positive antigen test results were false positives. In contrast, when prevalence increased to 10% (1,000 infected individuals per 10,000), the PPV rose to 81.6%. Despite identical test characteristics, merely changing the baseline prevalence completely altered the clinical interpretation of a positive test result. This example illustrates not only the importance of integrating prevalence, sensitivity, and specificity but also why Bayesian diagnostic reasoning remains highly counterintuitive, even when all relevant information is explicitly available.

### Cognitive biases and systematic errors

Despite being fundamental to evidence-based diagnostic reasoning and routinely taught throughout medical education, Bayesian diagnostic reasoning remains remarkably difficult even for experienced physicians. Early research demonstrated that individuals systematically misjudge PPVs in medical diagnostic scenarios while exhibiting high subjective certainty [6]. About 40 years ago, Eddy [7] highlighted this failure among physicians. Since then, numerous studies have shown that physicians consistently overestimate PPVs [8–10]. For instance, Gigerenzer et al. [8] reported that out of 160 gynecologists, 79% failed to correctly solve a Bayesian breast cancer diagnostic task. Collectively, these findings indicate that medical expertise alone does not reliably protect against Bayesian reasoning errors. These errors primarily originate during the mental representation of the task structure rather than the mathematical calculation itself. The most prominent (and intertwined) errors are the inverse fallacy and the base-rate neglect. The first relates to confusing a test’s sensitivity with its PPV [11]. The latter relates to underweighting or ignoring disease prevalence, which results in the belief that a positive test is nearly definitive, even for rare conditions [12, 13].

For many decades, a great body of research analyzed the cognitive processes behind these errors as well as instructional support to mitigate them. One of the most prominent and well-replicated results is the effect of the presentation format, specifically relative probabilities versus natural frequencies. The latter significantly improves the accuracy of probabilistic judgments (e.g., [14, 15–17]). To explain these effects, previous research has yielded two sets of theories. The ecological rationality account (ERA) focuses on how we humans are evolutionarily better adapted to process natural frequencies [18]. The nested sets account (NSA) focuses on how natural frequencies make a problem’s nested-set structure more salient, thereby leading to more analytical processing [18, 19].

However, the benefits of natural frequencies are neither universal nor sufficient to eliminate Bayesian reasoning errors. Their effectiveness depends on additional learner characteristics, including numeracy [20] and educational background [21]. Likewise, visual representations [16], nested-set visualizations [22], and linguistic reframing of questions to make the subsets more apparent [23] have all been shown to improve performance. Taken together, previous research has primarily focused on facilitating the correct representation of Bayesian problems by making their nested-set structure more transparent.

### The medical Bayesian performance score (MBPS)

Although Bayesian diagnostic reasoning has been investigated for several decades, performance is almost exclusively assessed dichotomously as either correct or incorrect. However, this approach cannot distinguish between fundamentally different reasons for an incorrect response. An incorrect response may simply reflect uncertainty or missing concepts. Alternatively, it may indicate a robust misconception—that is, an incorrect belief maintained with high subjective certainty. From an educational perspective, this distinction is important, as misconceptions are generally considered more resistant to instruction than missing knowledge and therefore require different educational interventions [24, 25]. Hence, combining correctness with subjective confidence has been recognized in misconception research to distinguish confidently held misconceptions from missing concepts [26, 27]. Building on this conceptual framework, we transferred this approach to Bayesian diagnostic reasoning.

Specifically, the present study introduces the Medical Bayesian Performance Score (MBPS), which combines participants’ diagnostic accuracy with their reported confidence in each response. It uses a scoring procedure previously developed to assess misconceptions about multimedia learning [26]. Rather than merely classifying responses as correct or incorrect, the MBPS enables a more differentiated interpretation of performance. High positive values indicate correct responses accompanied by high confidence. By contrast, high negative values reflect incorrect responses given with high confidence, a response pattern considered indicative of misconceptions. Finally, incorrect responses accompanied by low confidence are more consistent with uncertain or missing knowledge. Applying this framework to Bayesian diagnostic reasoning, we do not propose the MBPS as a replacement for conventional accuracy measures. Rather, it provides an additional perspective on the extent to which diagnostic errors are accompanied by subjective certainty.

### Individual differences in Bayesian diagnostic reasoning

Although systematic errors in Bayesian diagnostic reasoning are well established, considerably less is known about why some individuals perform substantially better than others do. The present study focuses on two explanatory factors: domain-specific knowledge/expertise and individual cognitive disposition.

First, physicians should benefit from their extensive medical knowledge and clinical expertise when interpreting diagnostic test results. However, as mentioned above, physicians frequently exhibit the same systematic errors as laypersons when solving Bayesian diagnostic tasks. Studies comparing different problem domains suggest that medical contexts may even evoke heuristic interpretations that interfere with analytical probability integration [21, 28]. Medical expertise itself also develops continuously throughout clinical practice. It thus remains unclear whether greater clinical expertise protects against persistent Bayesian reasoning errors.

Moreover, successful Bayesian reasoning may also depend on an individual’s tendency to engage in reflective rather than intuitive thinking. According to Dual-Process Theory, human cognition operates through two distinct mechanisms: Type 1 processes, which are rapid, automatic, and low-resource, and Type 2 processes, which are slower, deliberate, and reliant on working memory [29, 30]. While Type 1 intuition can be highly effective when grounded in extensive professional experience [31], it frequently introduces systematic biases in complex probabilistic tasks. Conversely, Type 2 processing can facilitate the rule-based integration of data and the overriding of erroneous intuitive judgments. However, deliberate reasoning does not necessarily lead to correct judgments, especially when it relies on insufficient knowledge or incorrect assumptions. The propensity to suppress an initial, compelling Type 1 response in favor of a reflective, analytical solution is defined as cognitive reflection, typically operationalized via the Cognitive Reflection Test (CRT; [30]).

In the context of Bayesian reasoning, deliberate Type 2 processing can facilitate the correct integration of probabilistic information, given that the underlying concepts are sufficiently understood. In their literature review, Barbey and Sloman [32] argue for a dual process model of judgment. Previous studies have consistently reported positive associations between cognitive reflection and Bayesian reasoning performance [18, 33, 34]. However, this relationship has primarily been investigated in student samples, leaving open the question of whether cognitive reflection similarly contributes to Bayesian reasoning within medical experts.

### The present study

The present study extends previous research on Bayesian diagnostic reasoning in two important ways. First, rather than assessing performance solely in terms of correct and incorrect responses, we applied the Medical Bayesian Performance Score (MBPS), a confidence-weighted measure designed to distinguish confidently held misconceptions from uncertain or missing concepts. Second, we investigated whether individual differences in Bayesian diagnostic reasoning are associated with medical knowledge, clinical expertise, and cognitive reflection. Based on previous evidence demonstrating the robustness of Bayesian reasoning errors, we expected misconceptions to be prevalent across all participant groups, resulting in generally low MBPS values. However, when exploring individual and professional variations, we formulated the following specific hypotheses:

- Hypothesis 1. Physicians will achieve higher MBPS values than the student control group, reflecting fewer misconceptions concerning medical Bayesian diagnostic problems.
- Hypothesis 2. Specialists and senior physicians will achieve higher MBPS values than resident physicians, indicating that clinical expertise could serve as a protective factor against misconceptions concerning medical Bayesian problems.
- Hypothesis 3. Cognitive reflection will be positively associated with MBPS values. In other words, participants with higher Cognitive Reflection Test (CRT) scores are expected to exhibit fewer misconceptions concerning medical Bayesian diagnostic problems.

## Methodology

### Participants and procedure

We planned our target sample size by aiming for at least 30 participants per group. This estimate is based on an a priori power calculation in G*Power to detect a medium-to-large effect (Cohen’s d = 0.65) with a power of 1 − β = 0.80 in a one-tailed *t* test at α = 0.05. Our final sample that provided informed consent and completed the study comprised *N* = 86 (51 female, 35 male; *M*_age_ = 30.95 years, *SD*_age_ = 13.01). It included a medical subsample of *N*_p_ = 30 physicians (9 female, 21 male; *M*_age_ = 44.23 years, *SD*_age_ = 12.39). We recruited the physicians from two local hospitals via a written study invitation, which briefly described the study and provided a link to the questionnaire. Participation was voluntary. The physicians worked in different medical specialties, including urology (*n* = 11), gynecology (*n* = 6), anesthesiology (*n* = 6), surgery (*n* = 4), and internal medicine (*n* = 1). Two physicians indicated another specialty without further specification. Furthermore, it included a student subsample of *N*_s_ = 56 undergraduate psychology and medical students (42 female, 14 male; *M*_age_ = 23.84 years, *SD*_age_ = 5.76). We recruited these students via our university’s online platform for research participants.

These subsamples enabled us to run group comparisons along two independent variables: (a) medical knowledge (physician group: *N*_p_ = 30 vs. student control group: *N*_s_ = 56) and (b) clinical expertise within the physician group (specialists and senior physicians: *N*_e_ = 19 vs. resident physicians: *N*_r_ = 11).

This study took place completely online using a web-based survey platform. The participants completed the following questionnaires in this order: demographics (age, sex, and professional status), topic interest, medical Bayesian problems (open-ended), Cognitive Reflection Test (CRT), and medical Bayesian problems (closed-ended). An English translation of the questionnaire content is provided as Additional file 1.

### Measures and instruments

#### Topic interest

We relied on four items based on Schiefele’s [35] emotional and value components to assess our sample’s interest in the topic “statistics and methodology.” The stem “I consider the topic ‘statistics and methodology’ as.” was followed by the attributes “boring” and “interesting” for the emotional component and “unimportant” and “useful” for the value component. Participants answered all four items on a Likert-type scale ranging from 1 (*fully disagree*) to 4 (*fully agree*). After reverse-scoring the negatively keyed items, the mean of all four items served as a measure of topic interest in statistics and methodology (Cronbach’s α = 0.76).

#### Medical Bayesian performance score (MBPS)

We used eight items to assess participants’ performance on medical Bayesian problems. Each item featured a medical diagnostic scenario, for instance, the aforementioned prostate cancer and SARS-CoV-2 scenarios. Each scenario featured a typical medical test with a given prevalence, specificity, and sensitivity. In all eight scenarios, statistical information was presented as probabilities expressed in percentages (rather than natural frequencies). We chose this format to reflect the common presentation of diagnostic test characteristics in clinical information materials. Participants were asked to estimate the probability that a patient is ill given a positive test result. The first four items were open-ended, requiring participants to state the probability themselves as a percentage. The other four items were closed-ended, allowing participants to choose from four predefined options. We chose to put the closed-ended items following the open-ended items to reduce priming effects.

For each of the eight items, participants also provided a subjective certainty rating (“How certain are you?”) on a Likert-type scale from 0 (*very uncertain*) to 4 (*very certain*). To combine correctness and subjective certainty, we adopted the scoring procedure developed by Eitel et al. [26], which was previously established in research on misconceptions [27].

First, we coded correct answers with + 1 and incorrect answers with − 1. Regarding the open-ended items, an answer was considered correct when it matched the value calculated using Bayes’ theorem, including minor rounding errors. We multiplied each correctness value (+ 1 or − 1) by the subjective certainty rating, yielding a range of − 4 to + 4. At the item level, higher positive values represent greater confidence in a correct answer, indicating “stronger certainty about correct knowledge.” More negative values, however, represent greater confidence in an incorrect answer, indicating response patterns consistent with misconceptions. Finally, values near zero indicate low confidence, suggesting the participants more or less guessed the answer, probably due to a lack of knowledge. Following Eitel et al. [26], we averaged the item scores to obtain an overall score. The mean of all item scores, excluding items with missing responses or confidence ratings, yielded the Medical Bayesian Performance Score (MBPS). It ranged from − 4 to + 4, with more negative values indicating stronger tendencies toward misconceptions and more positive values indicating greater confidence in correct knowledge (Cronbach’s α = 0.82).

#### Cognitive reflection test (CRT)

We administered the original Cognitive Reflection Test (CRT) by Frederick [30] to assess the participants’ degree of cognitive reflection. It comprised three open-ended items (bat-and-ball problem, machine problem, and water lily problem), each with a single correct solution, which is typically not the initially obvious intuitive answer. We coded correct answers with + 1 and incorrect answers with 0. The mean across all three items served as a measure of cognitive reflection, with higher values reflecting a stronger tendency toward reflective-analytical thinking (Cronbach’s α = 0.62).

### Statistical analyses

All statistical analyses were conducted using JASP and the alpha level was set at 0.05. For our specific hypotheses, we ran one-tailed *t* tests (Hypotheses 1 and 2) and a one-tailed Pearson’s correlation test (Hypothesis 3).

## Results

### Preliminary analyses

Table 1 provides descriptive statistics for all measures. We identified no statistically significant differences between our subsamples regarding interest in the topic “statistics and methodology”, *F*(2, 83) = 0.54, *p* = .585, or CRT performance, *F*(2, 83) = 0.11, *p* = .899.

**Table 1:** Descriptive statistics for all measures with means and standard deviations in parentheses

	Students *N*_s_ = 56	Physicians *N*_p_ = 30	Resident *N*_r_ = 11	Specialist/Senior *N*_e_ = 19	Total *N* = 86
Age	23.84 (5.76)	44.23 (12.39)	35.36 (6.42)	49.37 (12.18)	30.95 (13.01)
Topic interest^1^	2.72 (0.60)	2.83 (0.79)	2.96 (0.70)	2.75 (0.85)	2.76 (0.67)
MBPS^2^	−0.83 (1.13)	−0.55 (1.46)	−1.24 (0.97)	−0.15 (1.57)	−0.73 (1.26)
CRT^3^	0.65 (0.35)	0.61 (0.38)	0.61 (0.39)	0.61 (0.39)	0.64 (0.36)

^1^Mean score from 1 (lowest) to 4 (highest), ^2^Mean score from − 4 (lowest) to 4 (highest), ^3^Mean score from 0 (lowest) to 1 (highest)

Regarding the medical Bayesian problems, the percentages of incorrect answers ranged from 65.1% (56 of 86 participants; Item 6, Colorectal Cancer) to 93.0% (80 of 86 participants; Item 4, Corona). Item 4 was the most difficult, and none of the resident physicians answered it correctly. Generally, and as expected, incorrect response rates were lower for the closed-ended than for the open-ended items. This supported our decision to administer the closed-ended items after the open-ended tasks.

Concerning the MBPS, which combines correctness and certainty ratings, 74.4% of participants had values below zero for the most difficult item, Item 4. Remarkably, all groups, except the specialist/senior physicians, achieved negative scores on all items. These negative MBPS values reflected incorrect responses accompanied by higher confidence ratings. Table 2 shows each item’s scores for all groups.

**Table 2:** Percentages of incorrect answers (✗) and Medical Bayesian Performance Score (MBPS) with standard deviations in parentheses for each Bayes problem scenario

	Students *N*_s_ = 56		Physicians *N*_p_ = 30		Resident *N*_r_ = 11		Specialist/Senior *N*_e_ = 19		Total *N* = 86	
**Bayes Problem**	✗	MBPS	✗	MBPS	✗	MBPS	✗	MBPS	✗	MBPS
Prostate Cancer	80.4	−0.55 (1.82)	80.0	−0.38 (2.15)	90.9	−1.36 (1.75)	73.7	0.22 (2.18)	80.2	−0.49 (1.93)
Diabetes	92.9	−1.11 (1.56)	86.7	−0.63 (1.71)	100.0	−1.55 (1.93)	78.9	0.00 (1.86)	90.7	−0.95 (1.62)
Malaria	87.5	−1.04 (1.83)	83.3	−0.78 (1.95)	81.8	−1.10 (1.85)	84.2	−0.59 (2.03)	86.0	−0.95 (1.86)
Corona (PCR)	92.9	−1.18 (1.52)	93.3	−1.52 (1.45)	100.0	−1.82 (1.17)	89.5	−1.31 (1.62)	93.0	−1.29 (1.50)
Breast Cancer	91.1	−1.36 (1.24)	69.0	−0.67 (2.06)	81.8	−0.70 (2.11)	61.1	−0.65 (2.09)	83.5	−1.13 (1.58)
Colorectal Cancer	69.6	−0.63 (1.85)	56.7	−0.34 (1.95)	72.7	−1.27 (1.35)	47.4	0.22 (2.07)	65.1	−0.53 (1.88)
HIV	73.2	−0.37 (1.88)	80.0	−0.38 (1.98)	90.9	−0.89 (1.83)	73.7	−0.12 (2.06)	75.6	−0.38 (1.90)
Corona (Rapid Test)	73.2	−0.32 (1.88)	66.7	−0.11 (2.26)	81.8	−0.90 (1.91)	57.9	0.35 (2.37)	70.9	−0.25 (2.00)

### The effect of medical knowledge on the MBPS—Physicians versus students

In our first hypothesis, we assumed that physicians would have fewer misconceptions concerning medical Bayesian problems than the student control group and therefore obtain higher MBPS values. However, we could not detect a statistically significant MBPS difference between physicians and students, *t*(84) = 0.96, *p* = .169, *d* = 0.22, 95% CI [− 0.23, 0.66]. In the student group, 73.2% reached MBPS values below zero (indicating misconceptions), whereas 70.0% of the physicians showed negative values.

### The effect of clinical expertise on the MBPS—Expert physicians versus resident physicians

In our second hypothesis, we assumed that expert physicians (i.e., specialists and senior physicians) would exhibit misconceptions concerning medical Bayesian problems to a lesser extent than resident physicians would. Indeed, specialists and senior physicians scored significantly higher when solving the medical Bayesian problems than resident physicians, *t*(28) = 2.07, *p* = .024, *d* = 0.79 (large effect), 95% CI [0.01, 1.55]. Within the group of physicians, 100% of resident physicians had negative MBPS values, whereas only 52.6% of specialists and senior physicians did.

### The role of cognitive reflection

In our final hypothesis, we assumed that higher CRT performance would be associated with a lesser extent of misconceptions about medical Bayesian problems. We found a significant correlation between cognitive reflection and the performance on the medical Bayesian problems, *r* = .21, *p* = .026. Across all groups, higher CRT values were associated with higher MBPS values.

## Discussion

The present study investigated the influence of medical knowledge, clinical expertise, and cognitive reflection on performance in Bayesian diagnostic tasks. It took a first step toward integrating confidence-weighted assessment into research on Bayesian diagnostic reasoning. Specifically, we applied the Medical Bayesian Performance Score (MBPS), which combines response accuracy with subjective confidence. This allows us to distinguish response patterns consistent with confidently held misconceptions from uncertain or missing concepts.

Our interpretation of negative MBPS values follows the confidence-weighted scoring approach introduced by Eitel et al. [26]. In this approach, negative scores indicate that incorrect responses, weighted by subjective certainty, outweigh correct responses. Eitel et al. similarly interpreted negative mean scores as indicators of misconceptions. However, a negative mean score does not necessarily imply that all or most incorrect responses were given with high confidence. Moreover, although such response patterns are consistent with misconceptions, the MBPS does not directly assess the stability of underlying beliefs or systematic overconfidence.

Overall, our findings suggest that response patterns consistent with misconceptions concerning medical Bayesian problems were common across our sample. More specifically, practicing physicians did not differ significantly from the student control group in their average MBPS. Within the physician group, however, specialists and senior physicians achieved significantly higher MBPS values than resident physicians did. In addition, higher cognitive reflection was associated with higher MBPS values. Given the limited physician sample size, however, these findings should be considered preliminary.

### Medical knowledge does not predict MBPS

Contrary to our first hypothesis, practicing physicians did not achieve higher MBPS values than the student control group. This finding aligns with previous research demonstrating that even medical professionals systematically overestimate positive predictive values [7–9]. Descriptively, this pattern is highly pronounced in our sample: 73.2% (41 of 56) of students and 70.0% (21 of 30) of physicians obtained negative average MBPS values.

A particularly striking example was the SARS-CoV-2 diagnostic task, which proved to be the most difficult item in the entire test. Only 7.0% of participants answered it correctly, and none of the resident physicians identified the correct positive predictive value. This high prevalence of errors seems remarkable, given the clinical—and ultimately public health—decisions that depend on the correct interpretation of these test results.

The lack of a clear advantage for physicians over the control group suggests that medical knowledge alone may not be sufficient to overcome fundamental cognitive challenges in integrating diagnostic parameters.

One explanation is that these errors primarily arise during the mental representation of the problem rather than during the subsequent calculation. As proposed by Barbey and Sloman [32], successful Bayesian reasoning depends on correctly representing the nested-set structure underlying prevalence, sensitivity, and specificity. If individuals are unable to mentally process this structure, they are likely to rely on heuristic interpretations regardless of medical knowledge.

Our findings also contribute to the discussion on domain-specific effects in Bayesian reasoning. Previous studies with lay samples suggested that medical contexts may even activate background knowledge that interferes with analytical probability integration [28]. However, comparing physicians with a student control group revealed no evidence that formal medical knowledge itself provides a reliable advantage within medical Bayesian tasks.

Finally, the MBPS adds an educational perspective to these findings. By incorporating subjective certainty, it provides information about response patterns associated with misconceptions that cannot be obtained from accuracy scores alone. Consequently, frameworks from conceptual change research, which focus on identifying and remediating cognitive misconceptions, offer a highly valuable approach for interpreting and addressing these persistent errors [24, 25]. We will pick up this aspect in the section on future research.

### Clinical expertise is associated with higher MBPS

In contrast to the comparison between physicians and students, our second hypothesis was supported. In our sample, specialists and senior physicians achieved significantly higher MBPS values than resident physicians. This suggests that greater clinical expertise may be associated with fewer confidently held misconceptions in Bayesian diagnostic reasoning. Given the rather small physician sample, however, this finding should be considered preliminary.

This finding indicates that clinical expertise may play a more nuanced role than formal medical knowledge alone. While medical training appears insufficient to reliably reduce Bayesian reasoning errors, extensive clinical experience may gradually improve the integration of probabilistic information during diagnostic decision-making. One plausible explanation is that repeated exposure to diagnostic uncertainty and probabilistic data in daily clinical practice may—over time—sensitize clinicians to base-rate weights. While resident physicians might still focus too heavily on test-specific parameters (i.e., sensitivity and specificity), more experienced physicians may develop an implicit understanding of disease prevalence and its critical impact on positive predictive values. Alternatively, selection processes could account for this effect: individuals with higher analytical competence may be more likely to advance to senior professional positions. In this scenario, the observed MBPS difference would reflect baseline differences in cognitive skills rather than cumulative clinical expertise. Thus, the effect could stem from a partial overlap between experience and ability, meaning that clinical expertise might not only be the cause of better Bayesian reasoning but also a consequence of underlying analytical skills.

### Cognitive reflection predicts MBPS

Our third hypothesis was also supported. Higher scores on the Cognitive Reflection Test (CRT) were associated with higher MBPS values, indicating that participants with a stronger tendency toward reflective thinking exhibited fewer confidently held misconceptions in Bayesian diagnostic reasoning. This finding is consistent with dual-process theories of cognition, which propose that successful Bayesian reasoning requires overriding intuitive heuristic responses through deliberate analytical processing [30, 32]. Individuals with higher cognitive reflection may therefore be better able to inhibit intuitive—but misleading—judgments. Hence, they are more able to systematically integrate prevalence, sensitivity, and specificity to estimate positive predictive values. Importantly, this association was observed in a heterogeneous sample including students as well as physicians with different levels of clinical experience, suggesting that cognitive reflection may represent a broadly relevant factor in Bayesian diagnostic reasoning.

Furthermore, the observed correlation was modest, indicating that cognitive reflection explains only part of the variance in Bayesian diagnostic performance. This suggests that Bayesian reasoning errors cannot be attributed solely to individual differences in analytical thinking. Instead, they likely also reflect difficulties in constructing an appropriate mental representation of the underlying probabilistic relationships. Consequently, both cognitive reflection and conceptual understanding appear to contribute to successful Bayesian reasoning.

### Limitations and outlook for future research

First, data collection took place online and unsupervised. Although participants were explicitly instructed to complete the tasks independently and without external assistance, we cannot rule out that participants relied on external resources or tools. However, the generally low correctness rates among all groups give no reason to assume significant cheating during the tests.

Second, the sample size was modest, particularly within the physician subgroup (*N*_p_ = 30). After all, recruiting practicing physicians for empirical studies remains challenging because of their limited availability. Moreover, the comparison between resident physicians (*N*_r_ = 11) and specialists and senior physicians (*N*_e_ = 19) was based on particularly small subsamples. The small physician subsamples limit the precision of the estimated effect size, as reflected in its wide confidence interval. The statistical power of this comparison was limited. Consequently, our findings concerning clinical expertise should be considered preliminary and replicated in larger samples.

Third, although the MBPS is based on a previously established confidence-weighted scoring procedure [26], its application to medical Bayesian diagnostic reasoning is new. Despite its good internal consistency in the present study, additional research is required to scrutinize its construct validity, test–retest reliability, and applicability across different Bayesian reasoning tasks. In particular, future studies should examine whether negative MBPS values predict independently assessed misconceptions and their resistance to conceptual change.

Furthermore, the traditional Bayesian tasks we used for our MBPS represent highly simplified diagnostic decision processes. As noted by Mandel [36], these traditional Bayesian tasks do not fully capture realistic clinical contexts or multifaceted problem scenarios. After all, clinical decisions are embedded in complex environments involving large amounts of data and situational factors. Accordingly, our findings should not be interpreted as reflecting overall diagnostic competence but rather a specific aspect of probabilistic reasoning.

Finally, our student control group included a substantial proportion of psychology students, who are regularly exposed to statistics and research methodology. Their statistical training might have partially compensated for their comparatively limited medical knowledge. This might have reduced a potential difference between physicians and students. However, this explanation remains speculative because we did not directly assess statistical or medical knowledge. Furthermore, this group represents a particularly demanding comparison rather than a typical lay sample. At the same time, participation was voluntary, making some degree of self-selection likely. Individuals with greater interest in statistics may have been more willing to participate, suggesting that the observed prevalence of misconceptions may even represent a conservative estimate. Nevertheless, we could not detect statistically significant differences between our subsamples regarding interest in the topic “statistics and methodology.”

Despite these limitations, the present findings point to several promising directions for future research. In particular, the MBPS offers an opportunity to investigate Bayesian reasoning from the perspective of conceptual change research by distinguishing misconceptions from uncertain or missing concepts. Hence, it seems fruitful to address these misconceptions relying on evidence-based frameworks from conceptual change research. In particular, refutation texts that directly target misconceptions offer a promising approach [37, 38]. For example, medical students could first be confronted with the common misconception that a positive result on a highly sensitive diagnostic test necessarily indicates a high probability of having the disease. Then, this misconception could be explicitly challenged by an (extended) refutation text, which also explains the importance of disease prevalence and false-positive results. Finally, a worked example could illustrate the correct reasoning. Implementing such interventions in medical curricula could mitigate persistent failures in Bayesian diagnostic tasks and foster awareness of cognitive heuristics, even among expert clinicians.

## Conclusions

The present study introduces the Medical Bayesian Performance Score (MBPS) as a confidence-weighted approach to assessing Bayesian reasoning. By distinguishing confidently held misconceptions from missing concepts, the MBPS provides a complementary perspective to conventional accuracy measures and links research on Bayesian reasoning with conceptual change theory. Our findings add to the extensive literature demonstrating that Bayesian diagnostic reasoning remains challenging, even for medical professionals. Formal medical knowledge alone was not associated with higher MBPS values (and thus fewer confidently held misconceptions), whereas greater clinical expertise and cognitive reflection indeed were. Finally, these findings suggest that medical education should move beyond traditional statistical instruction and evaluate evidence-based conceptual change interventions, such as specific refutation-based training, as a means of improving Bayesian diagnostic reasoning.

## Supplementary Information

Additional file 1. This file provides an English translation of the questionnaire content used in the online survey.

## Data Availability

The data presented in this study are available from the corresponding author upon reasonable request.
