# Supplementary material for: Clinical expertise and cognitive reflection predict fewer misconceptions in Bayesian diagnostic reasoning
Source: BMC Med Educ. 2026 Nov 7;26:1806. doi: 10.1186/s12909-026-10764-z (PMC13744101; doi:10.1186/s12909-026-10764-z)
Supplement: Supplementary file 1 [file 12909_2026_10764_MOESM1_ESM.pdf]

Clinical expertise and cognitive reflection predict fewer misconceptions in Bayesian diagnostic reasoning

This document reproduces the English translation of the questionnaire content used in the online survey.

Section 1: Demographics

Age

Sex:

- Male
- Female
- No disclosure

Current educational status:

- Student
    - Psychology
    - Medicine
    - Other
- Resident physician
    - General Practice
    - Internal Medicine
    - Urology
    - Surgery
    - Other
- Specialist physician
    - General Practice
    - Internal Medicine
    - Urology
    - Surgery
    - Other
- Senior physician
    - General Practice
    - Internal Medicine
    - Urology
    - Surgery
    - Other

Section 2: Topic interest

I consider the topic ‘statistics and methodology’ as...

- Boring

Response scale from 1 (*fully disagree*) to 4 (*fully agree*)

- Interesting

Response scale from 1 (*fully disagree*) to 4 (*fully agree*)

- Unimportant

Response scale from 1 (*fully disagree*) to 4 (*fully agree*)

- Useful

Response scale from 1 (*fully disagree*) to 4 (*fully agree*)

Section 3: Open Ended Bayes Problems

Prostate Cancer

A PSA test is used for the early detection of prostate cancer. The PSA cutoff value—the level at which the test is considered positive—is set at 3 ng/ml. Let's assume that the probability of a 40-year-old man having prostate cancer is 0.1% (prevalence). The probability that the PSA test will yield a positive result (>3 ng/ml) in a man with prostate cancer is 85.9% (sensitivity). The probability that the PSA test will yield a negative result (<3 ng/ml) in a healthy man is 28.1% (specificity). Now, a 40-year-old man undergoes a screening examination and receives a positive PSA test result. How high do you estimate the probability that this man has prostate cancer?

How certain are you?

Response scale from 0 (*very uncertain*) to 4 (*very certain*)

Note: Correct answer 0.12%. Responses between 0.1% and 0.2% were scored as correct. Numbers adopted from MVZ-Dortmund [1].

Diabetes

The oral glucose tolerance test (oGTT) is used for the early diagnosis of diabetes mellitus. The sensitivity of the oGTT is 72%, meaning that in people with diabetes, the test yields a positive result 72% of the time. The specificity of the oGTT is 73%, meaning that in non-diabetics, the test will show no reaction—i.e., a negative result—with a probability of 73%. Approximately 1% of the population in Germany has diabetes (prevalence). Now, a randomly selected person takes the oral glucose tolerance test and receives a positive result. What do you estimate is the probability that this person has diabetes?

How certain are you?

Response scale from 0 (*very uncertain*) to 4 (*very certain*)

Note: Correct answer 2.62%. Responses between 2.5% and 3.0% were scored as correct. Numbers adopted from Griesel et al. [2].

Malaria

Some tropical hotels offer rapid tests for tourists to screen for malaria. The test is highly reliable for infected individuals and yields a positive result in 99% of cases (sensitivity). The test is also highly reliable for non-infected individuals and yields a negative result in 98% of cases (specificity). Let's assume that about 0.1% of tourists contract malaria (prevalence). Now, as a precaution, a tourist takes such a rapid test and receives a positive result. How high do you estimate the probability that this person has malaria?

How certain are you?

Response scale from 0 (*very uncertain*) to 4 (*very certain*)

Note: Correct answer 4.72%. Responses between 4.5% and 5.0% were scored as correct. Numbers adopted from M. Bühner (personal communication, October 2006).

Coronavirus (PCR)

PCR tests, which can detect viral RNA, are used to detect acute SARS-CoV-2 ("coronavirus") infection. Let's assume that the prevalence of acute coronavirus infection is 3%. The probability that a PCR test will yield a positive result in a person infected with the coronavirus is 98% (sensitivity). The probability that the PCR test will be negative in a person not infected with the coronavirus is 95% (specificity). Now, a randomly selected person has

received a positive result on the PCR test. How likely is it that this person is acutely infected with the coronavirus?

How certain are you?

Response scale from 0 (*very uncertain*) to 4 (*very certain*)

Note: Correct answer 37.74%. Responses between 35% and 40% were scored as correct. Numbers adopted from Schlenger [3].

Section 4: Cognitive Reflection Test (CRT)

The original three-item Cognitive Reflection Test was administered. Please see Frederick [4] for the original items.

Section 5: Closed Ended Bayes Problems

Breast Cancer

Mammography screening is an X-ray examination of the female breast used for the early detection of breast cancer. Let’s assume that the probability of a 40-year-old woman having breast cancer is 1% (prevalence). The probability that a mammogram will yield a positive result in a woman with breast cancer is 90% (sensitivity). The probability that a mammogram will yield a negative result in a healthy woman is 91% (specificity). Now, a 40-year-old woman participates in a breast cancer screening and receives a positive mammogram result. How high do you estimate the probability that this woman has breast cancer?

- 1%
- 10%
- 81%
- 90%

How certain are you?

Response scale from 0 (very uncertain) to 4 (very certain).

Note: Correct answer 10%; numbers adopted from Gigerenzer et al. [5].

Colorectal Cancer

The Hemoccult test is a test used to detect occult blood in the stool and is part of colorectal cancer screening. Suppose the probability that a 40-year-old man has colorectal cancer is 0.3% (prevalence). The probability that the Hemoccult test is positive in a man with colorectal cancer is 50% (sensitivity). The probability that the Hemoccult test is negative in a healthy man is 97% (specificity). A 40-year-old man now undergoes the Hemoccult test and receives a positive test result. How high do you estimate the probability that this man has colorectal cancer?

- 0.3%
- 3%
- 5%
- 50%

How certain are you?

Response scale from 0 (very uncertain) to 4 (very certain)

Note: Correct answer 5%; numbers adopted from Garcia-Retamero and Hoffrage [6].

Human Immunodeficiency Virus (HIV)

An ELISA test can detect certain immune proteins. It is used to diagnose Human Immunodeficiency Virus (HIV) infection. We estimate that approximately 0.1% of the population in Germany is HIV-positive (prevalence). The ELISA test yields a positive result in 99.9% of HIV-positive individuals (sensitivity). In 99.7% of uninfected test subjects, the ELISA test is negative (specificity). Now, a randomly selected person has a positive ELISA test result. How likely is it that this person is infected with HIV?

- 0.1%
- 25%
- 80%
- 99.7%

How certain are you?

Response scale from 0 (very uncertain) to 4 (very certain)

Note: Correct answer 25%; numbers adopted from Griesel et al. [2].

Coronavirus (Rapid Test)

Diagnostic antigen tests (“rapid tests”) can help identify people who are currently infected with SARS-CoV-2 (“coronavirus”). Let’s assume that 0.05% of those tested are actually currently infected with the coronavirus (prevalence). The probability that the rapid test will yield a positive result in a person infected with the coronavirus is 80% (sensitivity). The probability that the rapid test will yield a negative result in a person not infected with the coronavirus is 98% (specificity). A random person undergoes testing and receives a positive result. How likely is it that this person is currently infected with the coronavirus?

- 0.05%
- 2%
- 80%
- 98%

How certain are you?

Response scale from 0 (very uncertain) to 4 (very certain)

Note: Correct answer 2%; numbers adopted from Lein et al. [7].

References

1 MVZ-Dortmund. Prostataspezifisches Antigen (PSA), gesamt. 2026. https://www.medizin-zentrum-dortmund.de/de/laboratoriumsmedizin/untersuchungsprogramm/untersuchung/prostataspezifisches-antigen-psa-gesamt/
2 Griesel H, Gundlach A, Postel H, Suhr F. Elemente der Mathematik EDM: Einführungsphase Nordrhein-Westfalen. Schroedel; 2014.
3 Schlenger RL. PCR-Tests auf SARS-CoV-2: Ergebnisse richtig interpretieren [PCR Tests for SARS-CoV-2: Interpreting Results Correctly]. Deutsches Ärzteblatt. 2020;117:1194–5.
4 Frederick S. Cognitive reflection and decision making. J Economic Perspect. 2005;19:25–42. https://doi.org/10.1257/089533005775196732
5 Gigerenzer G, Gaissmaier W, Kurz-Milcke E, Schwartz LM, Woloshin S. Helping doctors and patients make sense of health statistics. Psychol Sci Public Interest. 2007;8:53–96. https://doi.org/10.1111/j.1539-6053.2008.00033.x
6 Garcia-Retamero R, Hoffrage U. Visual representation of statistical information improves diagnostic inferences in doctors and their patients. Soc Sci Med. 2013;83:27–33. https://doi.org/10.1016/j.socscimed.2013.01.034
7 Lein I, Leuker C, Antão E-M, von Kleist M, Jenny MA. SARS-CoV-2: Testergebnisse richtig einordnen [SARS-CoV-2: Interpreting test results correctly]. Deutsches Ärzteblatt. 2020;117:2304–5.
